# Satisfaction with treatment decision-making and long-term experiences of life-prolonging treatments for metastatic castration-resistant prostate cancer

**DOI:** 10.1186/s12904-026-02336-1

**Published:** 2026-09-19

**Authors:** Paula Routier Cañigueral, Agneta Wennman-Larsen, Sandra Doveson, Ulrika Rönningås, Maja Holm

**Affiliations:** 1 Department of Nursing Science, Sophiahemmet University, Valhallavägen 91 hus D, Box 5605, Stockholm, 114 86 Sweden; 2 Division of Insurance Medicine, Department of Clinical Neuroscience, Karolinska Institutet, Stockholm, Sweden; 3 Department of Health Care Sciences, Marie Cederschiöld University College, Stockholm, Sweden; 4 Department of Nursing, Umeå University, Umeå, Sweden; 5 Department of Oncology, Sundsvall County Hospital, Sundsvall, Sweden

**Keywords:** Prostatic neoplasm, Castration-resistant, Metastatic prostate cancer, Treatment decision-making, Treatment experiences, Person-centered care, Palliative care

## Abstract

**Background:**

Treatments in the phase of metastatic castration-resistant prostate cancer (mCRPC) aim to prolong survival and reduce the symptom burden. The fast development of life-prolonging therapies makes treatment decision-making (TDM) complex, weighting survival against possible side-effects and quality of life, especially in a real-world context. This study aimed to describe satisfaction with TDM at start of the first, second and third line of life-prolonging treatment and to describe and compare long-term treatment experiences when receiving one or several lines of treatment in patients with mCRPC.

**Methods:**

A longitudinal observational study. Participants completed a study-specific questionnaire every three months for up to two years from the start of any first line of life-prolonging treatment after mCRPC. Satisfaction with TDM was measured at the start of each new treatment line while treatment experiences were measured using the first and last completed questionnaire of each participant. Medical data was obtained from medical records. Descriptive statistics were used to present data while inferential statistics were used for analysis of group comparisons and over time changes.

**Results:**

A total of 122 participants (mean age 75.3 years) with an average follow-up time of 18.3 months were included. Satisfaction with TDM was generally high at the start of the first, second and third line of treatment, but lower regarding discussions on how treatment could affect their life situation. Treatment experiences were positive, yet there was a significant decrease from the first to the last follow-up in whether they would choose (*p* = 0.04) and recommend (*p* = 0.01) the same treatment to others. For participants who received one line of treatment, a significant decrease was found between the first and the last follow-up (*p* = 0.02), while no differences were found for those who received two or more lines.

**Conclusions:**

Despite high satisfaction with aspects of TDM and the treatment given it is important to discuss how the treatment can impact the patient’s life situation. The slightly decreased satisfaction over time on the treatment received could reflect a possible need to strengthen communication between patients and healthcare professionals, shifting from a disease-centered perspective towards a more person-centered and palliative care approach.

## Introduction

Treatment selection is one of the most complex but also most important decisions to make during a cancer trajectory [1]. Treatment decision-making (TDM) refers to the process by which patients and healthcare professionals evaluate available treatment options and jointly select the approach that best aligns with the patient’s clinical condition, preferences, values, and goals of care. Conversations regarding TDM occur in situations where multiple treatment scenarios are available and usually include the patient, a physician or oncologist, and sometimes significant others [2]. Treatment decisions usually present different challenges depending on the stage of the disease [3], patients’ personal values and goals [4–6] as well as their sociodemographic characteristics [2, 7]. These challenges are also faced by people diagnosed with prostate cancer (PC) [8]. In localised PC, treatment decisions entail therapies with curative intent such as prostatectomy and radiotherapy, or active surveillance [6, 9]. However, in metastatic PC (mPC), the intent of treatment is no longer curative but primarily palliative. It aims to control the disease by limiting the spread of the metastases, thereby reducing the symptom burden and improving quality of life, while also prolonging survival. Nevertheless, some life-prolonging treatments, defined in this study as therapies intended to extend survival and/or delay disease progression in advanced cancer, may be associated with undesired side-effects. Consequently, patients are often required to weigh the potential survival benefits against the impact of life-prolonging treatments on quality of life [10, 11].

In some cases, mPC will eventually progress to metastatic castration-resistant prostate cancer (mCRPC) [12]. In mCRPC, the cancer has spread to other parts of the body and cancer cells no longer respond to the initial hormone-suppressing treatment (castration resistance) [13]. The development of life-prolonging therapies for mCRPC over the past few decades has expanded the availability of treatments. These treatments include androgen receptor pathway inhibitors (ARPIs), chemotherapy, radiotherapy, and other systemic treatments each of which has demonstrated survival benefits while differing in side-effect profiles and routes of administration [14]. Despite ongoing life-prolonging treatment, disease progression may eventually be confirmed by imaging methods and/or increased blood levels of prostate-specific antigen (PSA) [15]. In such cases, patients may undergo multiple sequential lines of therapy, commonly referred to as first, second, and third line of treatments, if not more. Given the rapidity increasing number of treatment options for patients with mCRPC, there is currently a lack of information guiding decisions about the optimal sequence of treatments for these patients [16] which complicates the TDM process. As a result, patients remain dependent on the healthcare professionals’ preferences and experiences [15, 17, 18].

Demographic trends indicate an ageing population and rising life expectancy with a growing burden of PC [19], especially among older adults [20]. As the burden of PC continues to increase globally, people with mCRPC will also be exposed to longer periods of treatment with increasing palliative care needs. Although TDM has been extensively studied in localised [9, 21] and mPC [17, 22], there is a shortage of studies focusing on TDM and treatment experiences, in a real-world situation independent of the therapy chosen, in people with mCRPC. Understanding not only how satisfied they are with the TDM but also how they experience these treatments can help healthcare professionals and future patients in their treatment decisions. Therefore, this study aims to describe satisfaction with TDM at start of the first, second and third line of life-prolonging treatment for mCRPC. Another aim is to describe and compare long-term treatment experiences when receiving one or several lines of treatment.

## Methods

### Design and sample

The data used in this longitudinal observational study come from the multicentre, prospective PROCEED (PROstate Cancer – Experiences and Expectations During treatment) project [23, 24]. Four oncology departments were selected to represent different geographical regions in Sweden, both university and regional hospitals. Inclusion criteria for the PROCEED project were people about to start any first line of life-prolonging treatment with Androgen Reception Pathway Inhibitors (ARPIs), chemotherapy, radiotherapy or another systematic life-prolonging treatment after being diagnosed with mCRPC. All the eligible patients that fulfilled the inclusion criteria were identified by the local oncology team at each oncology department and consecutively invited to participate when about to start any first-line of life-prolonging treatment for mCRPC. Treatment assignments were made by the local oncology team independent of the study. The only exclusion criterion was inability to understand or express oneself in Swedish. A previous power analysis indicated that 120 patients would be sufficient to detect clinically meaningful changes in the Functional Assessment of Cancer Therapy-General (FACT-G), one of the instruments used in the overall PROCEED project, with 80% power at a 5% significance level [25, 26]. The target sample size was estimated to be between 120 and 150 patients, and the upper limit was appropriate to account for the expected attrition due to the participants’ poor health.

**Fig. 1:**
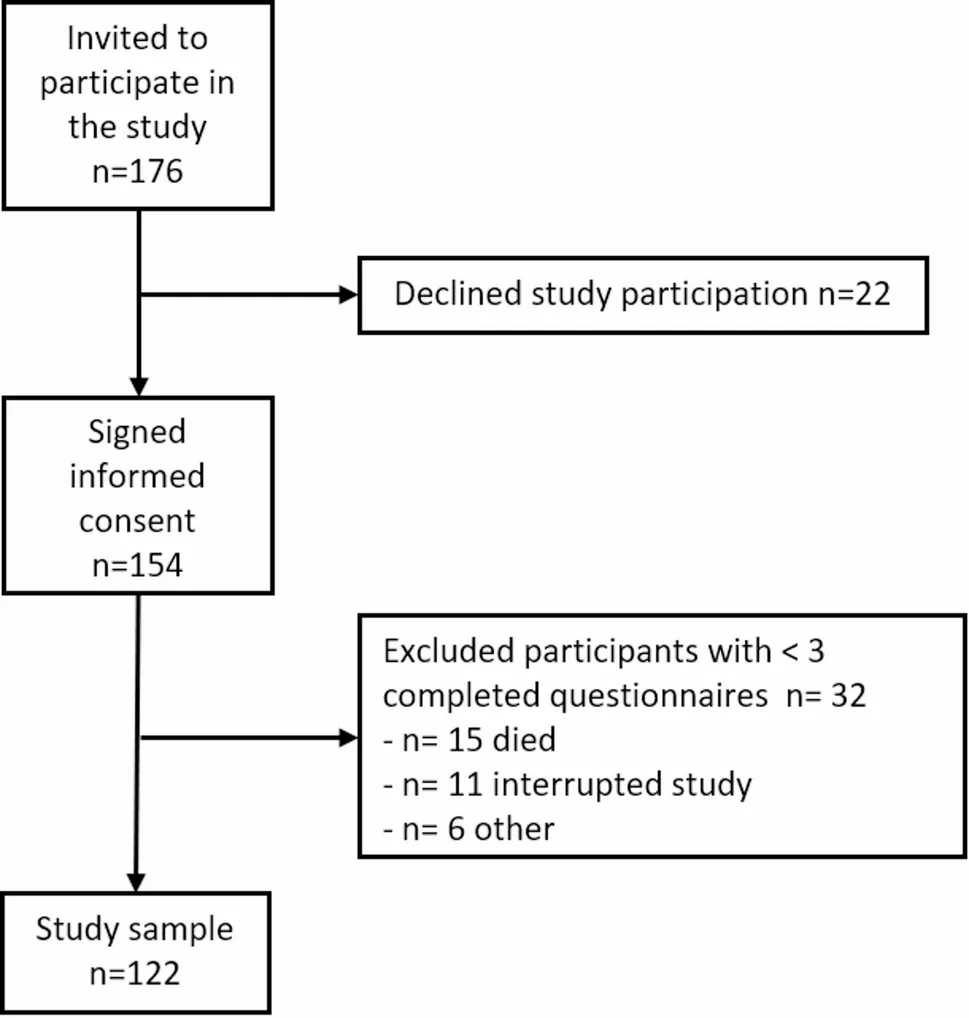
Flow chart of the study enrolment

Between April 2015 and March 2022, a total of 176 participants were invited to participate; 154 consented and were therefore included in the PROCEED study (Fig. 1).

For the present study, a subsample from the PROCEED study was used. The inclusion criterion was to have completed at least three study questionnaires (Fig. 1).

### Data collection

Data were collected using paper-based self-reported questionnaires. Questionnaires were administered at three different occasions: baseline, every three months during follow-up, and at the start of a new line of treatment. The baseline questionnaire was provided at the oncology department upon study inclusion. During the two-year follow-up period, subsequent questionnaires were distributed by post at approximately three-months interval by the research nurse of each oncology department. An additional questionnaire was sent when a new line of treatment was initiated. If a questionnaire was not returned, a reminder was sent by the research nurse. If the questionnaire was still not returned after the reminder, that follow-up assessment was considered missing and the next follow-up questionnaire was sent according to the three-month interval. Questionnaires that were returned after the two-year period (possibly due to deviations in the postal service) were also used if they did not exceed 26 months from inclusion.

The study outcomes (TDM and treatment experiences) as well as demographic data (marital status, education level and place of birth) were obtained from the self-reported questionnaires. Data such as age, date of death, medical data about the cancer diagnosis (PSA, tumour stage, node stage, metastasis stage, metastasis place and Gleason score) as well as lines of life-prolonging treatment received, were obtained from the participants’ medical records by the research nurse at each oncology department.

### Outcome measures

To measure satisfaction with TDM and treatment experiences, a study-specific instrument was used. At the start of the project, no validated instruments on TDM and treatment experiences were available in Swedish. Therefore, a study-specific instrument was developed in Swedish, inspired by pre-existing instruments available in English [27, 28]. The instrument was evaluated using think-aloud interviews with people with PC and was found to have good face validity [23, 29], the subscales also demonstrated good internal consistency, as described below.

*Satisfaction with TDM* was measured by 26 items divided into five domains (Physician communication, Nurse communication, Treatment staff communication, Technical competence, and Confidence and trust) at baseline and at the start of any new line of treatment. The items were translated into English and are presented in Table 1. The domains Physician and Nurse communication measured, e.g., the clarity of the information received, and the engagement perceived by the patients during treatment decision-making. Treatment staff communication, communication with healthcare professionals involved in the delivery and management of treatment including nurses and physicians, measured whether healthcare professionals discussed how the treatment could affect the participant’s life situation (housework, daily activities, relationships and emotions). Technical competence measured the participant’s perception of the healthcare professionals’ expertise and experience at treating the illness. Confidence and trust measured the overall trust in and confidence about the treatment process and healthcare professionals (Table 1). Each domain included several different items. The response options for each individual item were a 4-step Likert-type scale ranging from 0 *“No*,* not at all”* to 3 *“Yes*,* as much as I wanted”*. The total subscale score for each domain was calculated by summing all responses, multiplying the sum by the number of items in the scale and then dividing the product by the number of questions the participant had answered in the scale. At least 50% of the items in each subscale had to be completed to obtain a total subscale score. Physician communication consisted of 12 individual items with a total subscale score ranging between 0 and 36. Nurse communication and Technical competence consisted of three individual items each with a total subscale score ranging between 0 and 9. Treatment staff communication and Confidence and trust consisted of four individual items each with a total subscale score ranging from 0 to 12 (Table 1). Higher total subscale scores indicated higher satisfaction with TDM. The different domains demonstrated good internal consistency for all subscales [30], with a Cronbach’s alpha ranging from 0.73 to 0.95. Data on TDM from the questionnaires at the start of the first, second and third line of life-prolonging treatment were used in this study.

**Table 1:** Five domains to measure satisfaction with treatment decision-making with their respective items and score range.Three individual questions to measure treatment experiences

**Physician Communication (Score range 0–36)** Did your doctor(s) give explanations that you could understand?	**Nurse Communication (Score range 0–9)** Did your nurse(s) give explanations that you could understand?
Did your doctor(s) explain the possible benefits of your treatment?	Did your nurse(s) show genuine concern for you?
Did your doctor(s) explain the possible side effects or risks of your treatment?	Did your nurse(s) seem to understand your needs?
Did you have an opportunity to ask questions?	**Confidence and Trust (Score range 0–12)** Did you feel that the treatment staff answered your questions honestly?
Did you get to say the things that were important to you?	
Did your doctor(s) seem to understand what was important to you?	Did the treatment staff respect your privacy?
Did your doctor(s) show genuine concern for you?	Did you have confidence in your doctor(s)?
Did your doctor(s) seem to understand your needs?	Did you trust your doctor(s)’ suggestions for treatment?
Were you able to talk to your doctor(s) when you needed to?	**Technical Competence (Score range 0–9)** Did you feel your doctor(s) had experience treating your illness?
Were you encouraged to participate in decisions about your health care?	
Did you have enough time to make decisions about your health care?	Did you feel your doctor(s) knew about the latest medical developments for your illness?
Did your doctor(s) seem to respect your opinions?	Was the treatment staff thorough in examining and treating you?
**Treatment Staff Communication (Score range 0–12)** Did the treatment staff discuss how your health and treatment may affect your normal housework?	**Treatment experiences**
	Would you choose this treatment again? (No/Maybe/Yes)
Did the treatment staff discuss how your health and treatment may affect your normal daily activities?	Would you recommend this treatment to others with the same illness as you? (No/Maybe/Yes)
Did the treatment staff discuss how your health and treatment may affect your personal relationships?	On the whole, how would you rate this treatment? (0–4)
Did the treatment staff discuss how your health and treatment may affect you emotionally?	

*Treatment experiences* were measured by three individual questions answered during each follow-up when no treatment changes were initiated, approximately every three months after the baseline questionnaire. The first two, *“Would you choose this treatment again?” and “Would you recommend this treatment to others with the same illness as you?”* could be answered with “No”, “Maybe” or “Yes” whereas the last question *“On the whole*,* how would you rate this treatment?”* could be rated from 0 (poor) to 4 (excellent). These questions were treated as single items in the analysis and were obtained from each patient’s first and last completed questionnaire in this study. The questionnaires used consisted of the baseline questionnaire and the last completed follow-up questionnaire. If a baseline questionnaire was missing, the first and last completed follow-up questionnaire were used instead. To identify differences in treatment experiences depending on the number of lines of treatments received, the participants were separated into three groups: those who had received one, two, or three or more (≥ three) lines of life-prolonging treatment.

### Data analysis

Baseline characteristics are presented with descriptive statistics as frequencies (n) and percentages (%) for categorical variables, and as means (M) and standard deviations (SD) for continuous variables. Satisfaction with TDM is described using median (Mdn) and interquartile range (IQR) and treatment experiences are described using frequencies and percentages. Comparisons regarding sociodemographic and medical characteristics between the groups of participants receiving one, two, and ≥three lines of treatment were performed using one-way analysis of variance (ANOVA) with Tukey’s post-hoc test continuous variables. Chi-square (*X*^*2*^) for nominal variables and Kruskal-Wallis *H* test for ordinal variables [31]. To analyse differences in treatment experiences longitudinally between the first and the last follow-up, Wilcoxon Signed Rank Test was used [31]. The significance statistical level for all the inferential analyses was set at *p* < 0.05 using two-sided tests. The statistical analyses were performed with IBM SPSS software, version 29.0 (IBM Corp, Armonk, New York).

## Results

### Study sample

A total of 122 participants completed at least three study questionnaires and were therefore included in this study. The mean follow-up time between baseline and the last follow-up was 18.3 (SD 5.9) months (Table 2). Thirty-nine participants (32.0%) died during the two-year follow-up with a mean survival of 17.4 (SD 5.4) months from baseline. Among the participants who died during the two-year follow-up, there was a significant difference in time until death between those who received one line of treatment (14.8 months, SD 5.3), two lines of treatment (17.7 months, SD 4.0) and ≥three lines of treatment (21.5 months, SD 4.6) [*F* (2) = 6.265, *p* = 0.005] (Table 2).

**Table 2:** Time elapsed between baseline, when the study participants completed their last questionnaire (last follow-up), and death

Time elapsed		All patients (*n* = 122)	One line (*n* = 66)	Two lines (*n* = 33)	Three or more lines (*n* = 23)	*P* value*
Months from baseline to last follow-up	*n*	*122*	*66*	*33*	*23*	
	Mean (SD)	18.3 (5.9)	18.2 (6.3)	17.7 (5.9)	19.6 (4.7)	*= 0.396*
	Min-Max	(4–25)	(4–25)	(6–25)	(6–25)	
Months from baseline until death	*n*	*39*	*17*	*12*	*10*	
	Mean (SD)	17.4 (5.4)	14.8 (5.3)	17.7 (4.0)	21.5 (4.6)	*= 0.005*
	Min-Max	(8–28)	(8–25)	(11–25)	(11–28)	
Months from last follow-up until death	*n*	*39*	*17*	*12*	*10*	
	Mean (SD)	3.5 (2.8)	3.5 (2.6)	4.1 (3.8)	2.7 (1.6)	*= 0.528*
	Min-Max	(0–12)	(0–10)	(0–12)	(0–5)	

* Comparisons between the groups of participants receiving one, two, and ≥three lines of treatment were performed with one-way analysis of variance (ANOVA). Tukey’s post-hoc test was used to find specific group differences after finding an overall significant effect in ANOVA (not shown in the table)

### Demographic and medical characteristics

The mean age was 75.3 (SD 7.0) years at inclusion, and seven participants (5.7%) were younger than 65 years old (Table 3). Most participants were married or in a relationship (75.5%). A significant difference was found in age at baseline between the groups of participants who received one (M 77.7, SD 6.4), two (M 72.9, SD 6.5), and ≥three lines of treatment (M 72.3, SD 7.1) [*F* (2) = 9.077, *p* < 0.001]. Hence, the participants who received one line of treatment were a mean of 4.8 and 5.8 years older than those who received two (*p* = 0.002) and ≥three lines of treatment (*p* = 0.003), respectively. No other significant differences between the groups were detected for any of the demographic and medical variables (Table 3).

**Table 3:** General characteristics of study sample obtained at inclusion and at primary diagnosis of prostate cancer

Characteristics, at inclusion		All patients (*n* = 122)	One line (*n* = 66)	Two lines (*n* = 33)	Three or more lines (*n* = 23)	*P* value*
Age, years	Mean (SD)	75.3 (7.0)	77.7 (6.4)	72.9 (6.5)	72.3 (7.1)	*< 0.001*
	Min-max	(50–88)	(59–88)	(55–88)	(50–83)	
Marital Status, *n (%)*	Married/In a relationship	92 (75.5)	52 (78.8)	22 (66.7)	18 (78.3)	*= 0.241*
	Single/Widowed	26 (21.4)	11 (16.7)	11 (33.3)	4 (17.4)	
	Missing	4 (3.3)	3 (4.5)	0 (0)	1 (4.3)	
Education, *n (%)*	Elementary school	48 (39.3)	27 (40.9)	14 (42.4)	7 (30.4)	*= 0.192*
	High school	35 (28.7)	17 (25.8)	14 (42.4)	4 (17.4)	
	University	32 (26.2)	16 (25.4)	5 (15.2)	11 (47.8)	
	Other	3 (2.5)	3 (4.5)	0 (0)	0 (0)	
	Missing	4 (3.3)	3 (4.5)	0 (0)	1 (4.3)	
Place of birth, *n (%)*	Sweden	113 (92.6)	60 (90.9)	31 (93.9)	22 (95.7)	*= 0.491*
	Other European country	4 (3.3)	3 (4.5)	1 (3)	0 (0)	
	Country outside Europe	2 (1.6)	1 (1.5)	1 (3)	0 (0)	
	Missing	3 (2.5)	2 (3)	0 (0)	1 (4.3)	
PSA, *ng/mL*	Mean (SD)	68.9 (128.7)	44.1 (68.5)	85.7 (103.4)	116.0 (239.1)	*= 0.63*
	Min-max	(0.5–1141)	(0.5–461)	(0.8–425)	(4.6–1141)	

Characteristics, at primary diagnosis						*P* value*
Tumour stage, *n (%)*	T1	9 (7.4)	4 (6.1)	3 (9.1)	2 (8.7)	*= 0.852*
	T2	25 (20.5)	13 (19.7)	8 (24.2)	4 (17.4)	
	T3	62 (50.8)	37 (56.1)	15 (45.5)	10 (43.5)	
	T4	17 (13.9)	7 (10.6)	5 (15.2)	5 (21.7)	
	Tx	5 (4.1)	4 (6.1)	1 (3.0)	0 (0)	
	N/K	3 (2.5)	1 (1.5)	1 (3.0)	1 (4.3)	
	Missing	1 (0.8)	0 (0)	0 (0)	1 (4.3)	
Node stage, n *(%)*	N0	69 (56.6)	42 (63.6)	14 (42.4)	13 (56.5)	*= 0.333*
	N1	37 (30.3)	17 (25.8)	13 (39.4)	7 (30.4)	
	Nx	11 (9.0)	6 (9.1)	3 (9.1)	2 (8.7)	
	N/K	3 (2.5)	1 (1.5)	2 (6.1)	0 (0)	
	Missing	2 (1.6)	0 (0)	1 (3.0)	1 (4.3)	
Metastasis stage, n *(%)*	M0	66 (54.1)	35 (53)	17 (51.5)	14 (60.9)	*= 0.633*
	M1	52 (42.6)	30 (45.5)	14 (42.4)	8 (34.8)	
	Mx	1 (0.8)	1 (1.5)	0 (0)	0 (0)	
	Missing	3 (2.5)	0 (0)	2 (6.1)	1 (4.3)	
Metastasis place, n *(%)*	Bone	79 (64.8)	45 (68.2)	18 (54.5)	16 (69.6)	^*1*^
	Lymph nodes	36 (29.5)	18 (27.3)	14 (42.4)	4 (17.4)	
	Lung	3 (2.5)	2 (3.0)	0 (0)	1 (4.3)	
	Liver	1 (0.8)	0 (0)	0 (0)	1 (4.3)	
	Other	1 (0.8)	1 (1.5)	0 (0)	0 (0)	
	Missing	2 (1.6)	0 (0)	1 (3.0)	1 (4.3)	
Gleason Score, n *(%)*	6	11 (9.0)	8 (12.1)	2 (6.1)	1 (4.3)	*= 0.462*
	7	40 (32.8)	18 (27.3)	11 (33.3)	11 (47.8)	
	8	33 (27)	22 (33.3)	7 (21.2)	4 (17.4)	
	9	27 (22.1)	12 (18.2)	9 (27.3)	6 (26.1)	
	10	1 (0.8)	0 (0)	1 (3.0)	0 (0)	
	Missing	10 (8.4)	6 (9.0)	3 (9.1)	1 (4.3)	

*PSA* prostate-specific antigen

* Comparisons between the groups of participants receiving one, two, and ≥three lines of treatment were performed with one-way analysis of variance (ANOVA) for continuous variables, Chi-square (X^2^) for nominal variables and Kruskal-Wallis H test for ordinal variables. Tukey’s post-hoc test was used for the age variable to find specific group differences (not shown in the table)

^1^Too few participants in each group to do between-groups comparisons

**Fig. 2:**
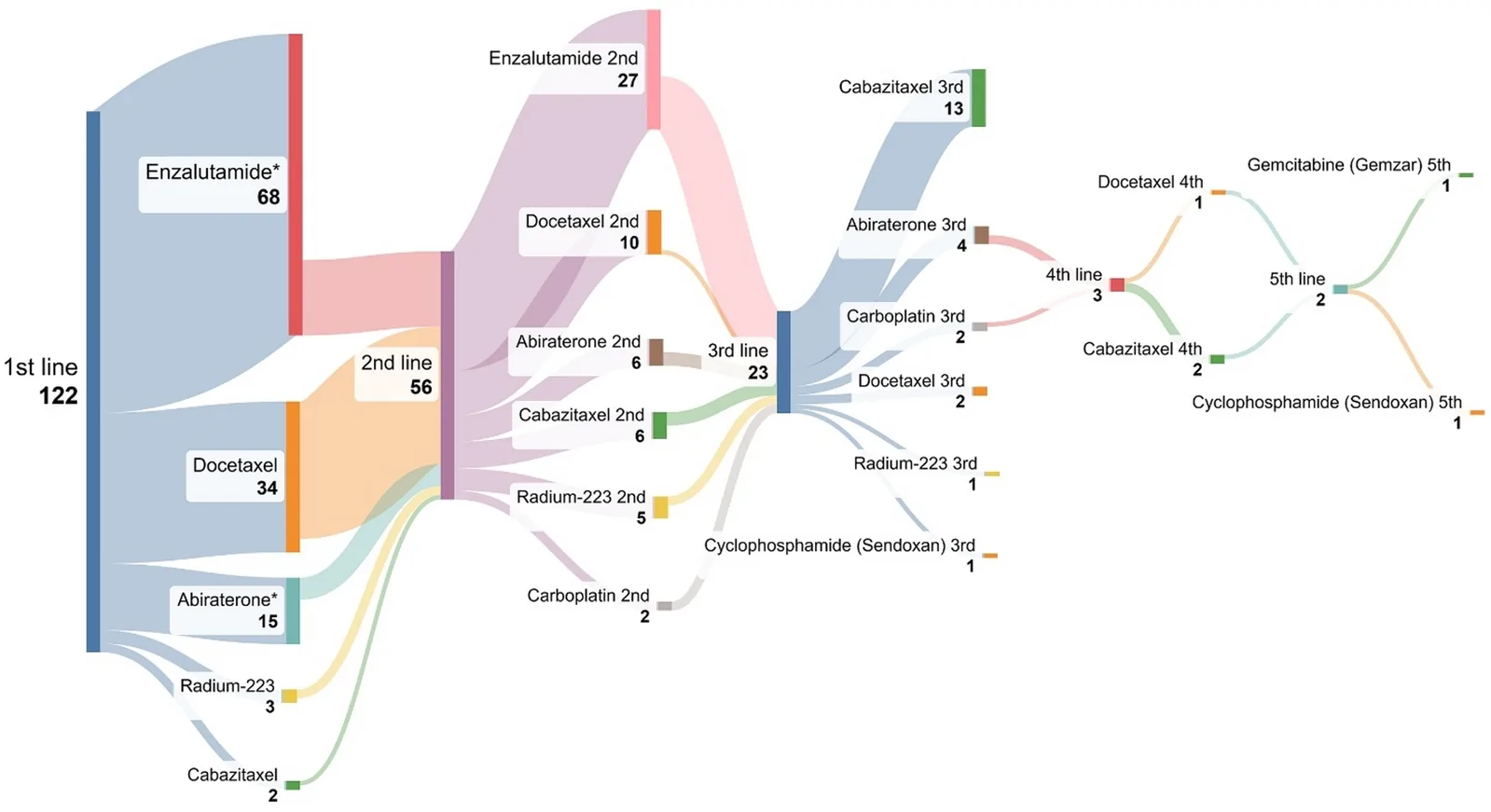
Therapies across different lines of treatment for the patients with mCRPC included in this study. *Two patients in each group had a combination with a study drug

Almost half of the participants (45.9%) received at least two lines of life-prolonging treatment during their time in the study (Fig. 2). Among them, 27.0% (*n* = 33) received two lines whereas 18.9% (*n* = 23) received ≥three lines of life-prolonging treatments. The most common first-line treatment was enzalutamide (55.7%) followed by docetaxel (27.9%) and abiraterone (12.3%). Enzalutamide (48.2%) and cabazitaxel (56.5%) were frequently given as second and third lines of treatment, respectively (Fig. 2).

### Treatment decision-making

The overall scores on satisfaction with TDM were high (Table 4). At the start of the first line of treatment, most participants rated high satisfaction with Physician communication (Mdn 34 out of 36 [IQR 30.6–36.0]), Nurse communication (Mdn 9 out of 9 [IQR 9.0–9.0]), Technical competence (Mdn 9 out of 9 [IQR 9.0–9.0]) and Confidence and trust (Mdn 12 out of 12 [IQR 11.0–12.0]). However, they rated lower satisfaction with Treatment staff communication, that concerns conversations about how the treatment would affect their life situation including normal housework, daily activities, personal relationships and their emotional state (Mdn 6 out of 12 [IQR 1.3–10.0]). The ratings remained stable in all domains with small variations at the start of the second and third line of treatment (Table 4).

**Table 4:** Satisfaction with TDM at the start of the first, second and third line of treatment

	Physician Communication	Nurse Communication	Treatment Staff Communication	Confidence and Trust	Technical Competence
Score range^1^	(0–36)	(0–9)	(0–12)	(0–12)	(0–9)
1st line of treatment (*n* = 122)					
* Median (IQR)*	34 (30.6–36.0)	9 (9.0–9.0)	6 (1.3–10.0)	12 (11.0–12.0)	9 (9.0–9.0)
* Missing*,* n (%)*	3 (2.5)	10 (8.2)	10 (8.2)	5 (4.1)	3 (2.5)
2nd line of treatment (*n* = 56)					
* Median (IQR)*	33.5 (24.8–36.0)	9 (8.0–9.0)	7 (0-9.8)	12 (10.7–12.0)	9 (8.0–9.0)
* Missing*,* n (%)*	8 (14.3)	9 (16.1)	8 (14.3)	9 (16.1)	8 (14.3)
3rd line of treatment (*n* = 23)					
* Median (IQR)*	33 (27.0–36.0)	9 (8.8-9.0)	7 (2.5–11.0)	11.5 (9.8–12.0)	9 (7.0–9.0)
* Missing*,* n (%)*	4 (17.4)	5 (21.7)	1 (4.3)	5 (21.7)	19 (17.4)

*TDM* Treatment decision-making

^1^Minimum and maximum values of the total subscale score

### Treatment experiences

Overall, the participants responded positively to the questions about their treatment experiences: whether they would choose the same treatment again, recommend the treatment to others and how they would rate it on the whole (Table 5).

**Table 5:** Treatment experiences measured at the first and last follow-up

			First follow-up						Last follow-up						Total	*p* value*
			No		Maybe	Yes		Missing	No		Maybe	Yes		Missing		
*“Would you choose * *this treatment again?”*	All	N (%)	2 (1.6)		20 (16.4)	90 (73.8)		10 (8.2)	6 (4.9)		27 (22.1)	80 (65.6)		9 (7.4)	122 (100)	*0.038*
	One line	N (%)	1 (1.5)		4 (6.1)	57 (86.4)		4 (6.1)	4 (6.1)		10 (15.2)	47 (71.2)		5 (7.6)	66 (100)	*0.022*
	Two lines	N (%)	1 (3.0)		9 (27.3)	19 (57.6)		4 (12.1)	2 (6.1)		10 (30.3)	18 (54.5)		3 (9.1)	33 (100)	*0.508*
	≥Three lines	N (%)	0 (0)		7 (30.4)	14 (60.9)		2 (8.7)	0 (0)		7 (30.4)	15 (65.2)		1 (4.3)	23 (100)	*1.000*
*“Would you recommend this treatment* * to others with the same illness as you?”*	All	N (%)	1 (0.8)		18 (14.8)	90 (73.8)		13 (10.7)	2 (1.6)		30 (24.6)	80 (65.6)		10 (8.2)	122 (100)	*0.021*
	One line	N (%)	0 (0)		8 (12.1)	51 (77.3)		7 (10.6)	1 (1.5)		12 (18.2)	47 (71.2)		6 (9.1)	66 (100)	*0.146*
	Two lines	N (%)	1 (3.0)		5 (15.2)	23 (69.7)		4 (12.1)	1 (3.0)		10 (30.3)	19 (57.6)		3 (9.1)	33 (100)	*0.289*
	≥Three lines	N (%)	0 (0)		5 (21.7)	16 (69.6)		2 (8.7)	0 (0)		8 (34.8)	14 (60.9)		1 (4.3)	23 (100)	*0.453*

			Poor	Fair	Good	Very Good	Excellent	Missing	Poor	Fair	Good	Very Good	Excellent	Missing	Total	*p* value*
*“On the whole, how would you rate this treatment?”*	All	N (%)	0 (0)	12 (9.8)	23 (18.9)	46 (37.7)	32 (26.2)	9 (7.4)	1 (0.8)	16 (13.1)	27 (22.1)	37 (30.3)	32 (26.2)	9 (7.4)	122 (100)	*0.525*
	One line	N (%)	0 (0)	3 (4.5)	15 (22.7)	23 (34.8)	21 (31.8)	4 (6.1)	1 (1.5)	7 (10.6)	15 (22.7)	18 (27.3)	20 (30.3)	5 (7.6)	66 (100)	*0.617*
	Two lines	N (%)	0 (0)	5 (15.2)	6 (18.2)	11 (33.3)	8 (24.2)	3 (9.1)	0 (0)	6 (18.2)	6 (18.2)	11 (33.3)	7 (21.1)	3 (9.1)	33 (100)	*0.607*
	≥Three lines	N (%)	0 (0)	4 (17.4)	2 (8.7)	12 (52.2)	3 (13.0)	2 (8.7)	0 (0)	3 (13.0)	6 (26.1)	8 (34.8)	5 (21.7)	1 (4.3)	23 (100)	*1.000*

* Comparisons between the first and the last follow-up were made using Wilcoxon Signed Rank Test

#### Preferences for choosing the same treatment again

For the question whether they would choose the same treatment again or not, there was a statistically significant decrease in the proportion of participants who were positive to choosing the same treatment between the first (73.8%) and the last (65.6%) follow-up (*Z* = − 2.092, *p* = 0.04) (Table 5). For the participants who only received one line of treatment, a statistically significant difference was also found between the first (86.4%) and the last (71.2%) follow-up (*Z* = -2.219, *p* = 0.02), while no significant differences were found between the first and last follow-up for those who received two (*p* = 0.51) or ≥three (*p* = 1.00) lines of treatment (Table 5).

#### Willingness to recommend the same treatment

For the question if the participants would recommend the treatment to others with the same illness, a statistically significant higher proportion answered “Yes” at the first (73.8%) than at the last (65.6%) follow-up (*Z* = -2.309, *p* = 0.02). No statistically significant differences in proportions could be seen between the first and the last follow-up when comparing the groups that received one (*p* = 0.15), two (*p* = 0.29), or ≥three lines of treatment (*p* = 0.45) (Table 5).

#### Ratings of the treatment on the whole

Most of the participants rated the treatment as “Very good” or “Excellent” both during the first and the last follow-up. The actual proportions were “Fair” (9.8% vs. 13.1%), “Good” (18.9% vs. 22.1%), “Very good” (37.7% vs. 27.3%), and “Excellent” (26.2% vs. 26.2%). There were no statistically significant differences between the two follow-ups, neither for the overall sample (*p* = 0.53) nor for each group depending on the number of lines of treatments received (Table 5).

## Discussion

The participants in this study with incurable prostate cancer and potential palliative care needs generally gave high scores for satisfaction with TDM and treatment experiences. Nevertheless, they were less satisfied on conversations regarding how the treatment could affect their life situation and with a growing uncertainty over time on whether they would choose the same treatment again or recommend the treatment to others. Apart from that, the positive treatment experiences were consistent over time between groups receiving one, two, or ≥three lines of treatment.

The results indicate that TDM experiences among the participants were positive and satisfactory in terms of communication, confidence and trust and technical competence. Open discussions and patient engagement in decision-making processes during incurable cancers has previously been shown to contribute to satisfaction with how the treatment decisions are made [32]. These findings are also in accordance with previous results from the PROCEED project, using data from the first year of follow-up, in which patients reported high levels of satisfaction with the explanations and encouragement they received from healthcare professionals concerning participation in TDM [26]. The participants in the present study rated high levels of satisfaction with trust in the healthcare professionals and satisfaction with the information received. However, the results obtained also show that participants rated lower satisfaction with conversations about how the treatment could affect their life situation or, in some cases, were not even offered an opportunity to discuss this topic. This is important to discuss at this stage of the disease since the treatment aims to alleviate symptoms and improve quality of life, but also may come with some side-effects possibly affecting several aspects of the patients’ life. Healthcare professionals have a responsibility to assist patients in making informed decisions [18], and their recommendations are important in helping patients decide whether to accept or decline a treatment [33]. Nevertheless, selecting the most appropriate treatment for an individual patient in a real-world setting can be challenging due to the availability of multiple life-prolonging treatments and the possibility for several sequential lines of therapy with differing benefits and risks [34, 35]. In addition, treatment decisions must be tailored to patient’s clinical characteristics, preferences and treatment goals. These challenges may explain the findings of the present study, which suggest insufficient communication between the patient and the healthcare professional about how their disease and treatment could affect their emotional well-being, everyday functioning, and relationships. Such communication gap may have implications for the patients’ satisfaction with the treatment decision process and the treatment received.

Almost 80% of the participants rated the treatment they received from good to excellent both during the first and the last follow-up. Still, a significant decrease over time in whether the participants would choose the treatment again or recommend it to others was found. These findings may suggest that the participants expected the treatments received to have a better and/or longer-lasting effect than they had while understanding that there may experience some side-effects [36]. Unmet expectations on the treatments received may impact negatively on their treatment experiences. However, considering the advanced stage of the disease in this study population, a decline in their physical and emotional well-being could also partly explain the observed decrease in treatment satisfaction.

When separated into groups depending on the number of lines of treatments received, only the patients who received one line of treatment presented a significant decrease over time in their ratings on whether they would choose the same treatment again. Those patients were also older and lived for a shorter time with the disease than patients receiving further lines of treatment. It could be argued that earlier undesired symptoms during the cancer trajectory and unfitness for, or unwillingness to, undergo further therapies may be an explanation to less satisfying treatment experiences. Older adults with mPC usually put greater emphasis on patient-reported outcomes such as quality of life than on survival measurements [37]. However, treatment conversations with older adults may not always be person-centered or based on frailty assessment [38]; instead they may focus on cancer-specific factors [39] or chronological age, which can be considered contrary to a palliative care approach [40]. Moreover, it is not unusual to continue life-prolonging treatments by default, without considering a permanent discontinuation [41], possibly leading to overtreatment [42]. This practice may not benefit older adults with mCRPC, who tend to receive less specialized palliative care than their younger counterparts when facing advanced cancer [43]. Frailty assessments could reveal vulnerabilities linked to physiological age, potentially leading to the need to discontinue treatment [44]. Therefore, the slight decline in how positively the older patients rated their treatment may be attributed to age-related vulnerabilities that were not sufficiently addressed during treatment conversations or to the lack of a palliative care approach that focused on the needs rather than the prognosis. These findings may highlight the need to strengthen communication between patients and the healthcare professionals and to adopt a more person-centred approach, including the early integration of palliative care and, when appropriate, geriatric assessments in treatment conversations.

### Strengths and limitations

To our knowledge, this is the first study assessing TDM and treatment experiences in mCRPC depending on the number of lines of treatment received during a two-year period, regardless of the specific life-prolonging treatment. The study includes a real-world and comparatively large sample considering the frailty and life-limiting disease stage of the study group [45]. Utilising real-world data by not excluding those with comorbidities gives a more accurate and reliable representation of the study population. Even though the sample size was relatively large given the participants’ health status, it was too small to enable certain subgroup analyses, and the comparisons between groups should therefore be interpreted with caution. Similarly, it must be considered that the statistical significances found in the results may not be clinically relevant. A strength is the multicentre representation of both university and regional hospitals across different regions of Sweden. Nevertheless, the sample is limited to four sites and the outcomes of this study, especially TDM, may be context bound. Therefore, the representativeness of the findings should be exercised with caution when generalizing the results to the wider population of patients with mCRPC, especially outside Sweden. A limitation is also that the participants needed to speak and understand Swedish, possibly restricting the participation of individuals of other ethnicities. Since effective communication and understanding of patients’ needs are crucial aspects of treatment decisions, the results do not adequately represent non-Swedish-speaking individuals.

Another limitation is the use of a self-developed instrument, which limits the generalisability and comparability of the data with other populations and research due to the limited psychometric testing and validation. However, the instruments were evaluated, showing good face validity and high Cronbach’s alpha in the TDM domains, indicating that the subscales had high consistency.

## Conclusions

The longer patients live with mCRPC, the more decisions need to be made about whether to continue with further lines of life-prolonging treatment or not, considering possible treatment benefits, side-effects, patient preferences and palliative care needs. Even if the participants were generally satisfied with different aspects of TDM and with the treatment given, this study suggest a need to strengthen communication between patients and the healthcare professionals regarding how treatment may affect different aspects of their lives. This could be achieved by shifting from a disease-centered perspective towards a more person-centered and palliative care approach.

Future research should further explore satisfaction in the context of multiple lines of treatment and age-related vulnerabilities, as well as evaluating if treatment satisfaction is sustained over time. Moreover, to facilitate a more comprehensive assessment of satisfaction with TDM and the treatments received, there is a need for further research using developed and validated instruments, specifically designed for this population.

## Data Availability

The data supporting the findings of this study are not publicly available due to sensitivity concerns and Swedish ethical and data protection regulations. Readers may contact Professor Agneta Wennman-Larsen (agneta.wennman-larsen@shh.se) for inquiries regarding the data.

## Abbreviations

mPC: Metastatic prostate cancer
mCRPC: Metastatic castration-resistant prostate cancer
TDM: Treatment decision-making
PSA: Prostate-specific antigen
